# Myocardial injury and long-term oncological outcomes in patients undergoing surgery for colorectal cancer

**DOI:** 10.1007/s00384-023-04528-0

**Published:** 2023-09-19

**Authors:** Jawad Ahmad Zahid, Adile Orhan, Noor Al-Huda Hadi, Sarah Ekeloef, Ismail Gögenur

**Affiliations:** 1 Center for Surgical Science, Department of Surgery, Zealand University Hospital, University of Copenhagen, Lykkebækvej 1, 4600 Køge, Copenhagen, Denmark; 2 Department of Clinical Medicine, University of Copenhagen, Copenhagen, Denmark

**Keywords:** Myocardial injury after noncardiac surgery, Colorectal cancer, Surgical oncology, Overall survival, Cancer recurrence, Disease-free survival

## Abstract

**Purpose:**

Myocardial injury after noncardiac surgery (MINS) is associated with increased mortality and postoperative complications. In patients with colorectal cancer (CRC), postoperative complications are a risk factor for cancer recurrence and disease-free survival. This study investigates the association between MINS and long-term oncological outcomes in patients with CRC in an ERAS setting.

**Methods:**

This retrospective cohort study was conducted at Zealand University Hospital, Denmark, between June 2015 and July 2017. Patients undergoing CRC surgery were included if troponin was measured twice after surgery. Outcomes were all-cause mortality, recurrence, and disease-free survival within five years of surgery.

**Results:**

Among 586 patients, 42 suffered MINS. After five years, 36% of patients with MINS and 26% without MINS had died, p = 0.15. When adjusted for sex, age and UICC, the hazard ratio (aHR) for 1-year all-cause mortality, recurrence, and disease-free survival were 2.40 [0.93–6.22], 1.47 [0.19–11.29], and 2.25 [0.95–5.32] for patients with MINS compared with those without, respectively. Further adjusting for ASA status, performance status, smoking, and laparotomies, the aHR for 3- and 5-year all-cause mortality were 1.05 [0.51–2.15] and 1.11 [0.62–1.99], respectively. Similarly, the aHR for 3- and 5-year recurrence were 1.38 [0.46–4.51], and 1.49 [0.56–3.98] and for 3- and 5-year disease-free survival the aHR were 1.19 [0.63–2.23], and 1.19 [0.70–2.03].

**Conclusion:**

In absolute numbers, we found no difference in all-cause mortality and recurrence in patients with and without MINS. In adjusted Cox regression analyses, the hazard was increased for all-cause mortality, recurrence, and disease-free survival in patients with MINS without reaching statistical significance.

## Introduction

Improvements in diagnostics, screening, and treatment have led to an increase in survival after colorectal cancer in the past decades [1]. In the same time period, the development and use of the Enhanced Recovery After Surgery (ERAS) protocol has been established [2, 3]. The ERAS protocol consists of evidence-based guidelines that include a multimodal approach to preoperative, intraoperative, and postoperative care, including surgical, anesthetic, and nursing recommendations. The ERAS protocol is associated with improved recovery and reduced complications after surgery [3, 4]. Nonetheless, a recent global study reports that postoperative complications occur in more than 45% of patients with colorectal cancer [5] and in Denmark, more than 20% do not survive the first five years after surgery [6]. Cardiovascular complications including myocardial infarction, congestive heart failure, and stroke are responsible for a significant number of deaths during surgery, making up for at least one-third of the total [7–9]. Knowledge about the occurrence and consequences of myocardial injury after noncardiac surgery (MINS) in an ERAS setting is sparse. MINS is most commonly defined as at least one elevated postoperative cardiac troponin due to a presumed ischemic event [9–11]. In patients undergoing colorectal cancer surgery in an ERAS setting, MINS is seen in approximately 7% of patients and is associated with postoperative complications [12]. Moreover, postoperative troponin levels among patients undergoing noncardiac surgery are associated with both increased short- and long-term mortality [8, 10, 13]. In surgical patients with colorectal cancer, postoperative complications are independent risk factors for all-cause mortality, disease-free survival and worsen the risk of cancer recurrence [14]. Interestingly, clinical studies have shown an association between cancer and cardiovascular morbidity [15, 16]. The aim of this study was thus to estimate the effect of MINS during the first seven days after surgery for colorectal cancer on long-term all-cause mortality, recurrence, and disease-free survival in an ERAS setting.

## Materials & methods

### Study design, data sources, and ethics

We performed a retrospective cohort study including adults undergoing elective surgery for colorectal cancer at Zealand University Hospital, Denmark, between June 2015 and July 2017. In this period, troponin I was scheduled to be measured daily on all patients undergoing surgery in the department until discharge as part of the screening for an international multicenter randomized controlled trial, the MANAGE study [17]. However, due to either early discharge of the patient or missed collection of blood samples, not all patients had two or more troponin I measurements [12]. All patients were treated in accordance with existing national guidelines. Clinical care at the Department of Surgery follows the ERAS society guidelines to ensure an optimal perioperative course [18]. The local ERAS protocol has been described in detail previously and includes elements such as patient education, using minimally invasive techniques, and early mobilization [19–21].

Patients whose troponin levels were measured twice within seven days of surgery were included. Data were obtained from electronic patient records and included patient demographics, comorbidities, medication, WHO performance status [22], Revised Cardiac Risk Index (RCRI) [23], Preoperative Score to Predict Postoperative Mortality (POSPOM) score [24], and treatment characteristics including details regarding the surgery, anesthesia, length of hospitalization, intensive care unit stay, and postoperative complications as defined in a previous paper [12]. As part of a quality assurance study at Department of Surgery at Zealand University Hospital, five-year follow up were conducted on patients and according to Danish Legislation no ethical approval was required. The study was reported according to the Strengthening the Reporting of Observational Studies in Epidemiology (STROBE) statement.

### Exposure of interest

The exposure of interest was the postoperative occurrence of MINS. It was defined in accordance with the MANAGE trial and has been described thoroughly in previous publications [12, 17]. The Department of Clinical Biochemistry at Zealand University Hospital measured cardiac troponin I and in the study period, they used the Siemens Healthcare Dimension Vista^®^ (Siemens, Munich, Germany) with the LOCI^®^ cardiac troponin I assay. The limit of detection of troponin I for this assay was 15 ng per liter with a cut-off ≥ 45 ng per liter for cardiac ischemia (99^th^ percentile for the upper reference limit for mean troponin from a normal reference population, 10% coefficient of variation at 40 ng per liter) [25].

### Outcome of interest

Our outcomes of interest were all-cause mortality, recurrence and disease-free survival. All outcomes were investigated at postoperative year one, three, and five. All-cause mortality was defined as patients not alive at the respective time points. Recurrence was defined as either a local recurrence or a remote metastasis appearing more than 180 days after surgery. Metastases found within 180 days after surgery were interpreted as undetected metastases at the time of surgery and thus not classified as recurrence. Disease-free survival was defined as no occurrence of either recurrence or death in the specified follow-up period.

### Statistical methods

Continuous data were expressed as mean (standard deviation) and categorical data as numbers with percentages. Patient data were analyzed using either the Pearson’s Chi-squared test or Fisher’s exact test for dichotomous variables and the Student’s t-test or Wilcoxon rank-sum test for continuous data. All-cause mortality, recurrence, and disease-free survival were examined by the Kaplan–Meier estimator, including the log-rank test and Cox regression models. Unadjusted and adjusted cox regression models were performed. Results were expressed as hazard ratios (HR) with 95% confidence intervals. The one-year model was adjusted for sex, age groups (below 70 or 70 and above), and UICC stages (1 and 2 or 3 and 4), whereas the three- and five-year models additionally were adjusted for ASA status (1 and 2 or 3 and 4), WHO performance status (0, 1, 2 or above), smoking status (never smoked, previous smoker, and current smoker), and laparotomies. The variables were chosen based on clinical hypotheses focusing on adjusting for underlying bias such as age and sex and disease severity. The age cut-off was based on guidelines from Danish Colorectal Cancer Group regarding the adjuvant therapy. In the cox regression model, the Schoenfeld test were used to determine if the models fulfill the proportionality assumption. The models were adjusted for a maximum of ten events per variable in order to avoid overfitted models [26, 27]. The method proposed by Fine and Gray were performed for competing risk analyses [28]. The statistical analyses were performed using SAS 9.4.

## Results

A total of 586 patients with five-year follow-up were included in our study, of which 42 patients suffered MINS within 7 days of surgery. Patients in the MINS group were significantly older, had a lower proportion of non-smokers, and higher ASA score, and higher WHO performance status. The patients with MINS had more comorbidities, but there was no difference in cancer stages nor in the pre-surgical and surgical treatment or procedures. The MINS group experienced more postoperative complications including a longer length of stay and intensive care unit stays. Baseline and perioperative data are presented in Table 1.

**Table 1:** Baseline data on surgical patients with and without myocardial injury

	**MINS** **n = 42**	**No MINS** **n = 544**
**Demographics**		
Male	21 (50%)	305 (56%)
Age		
• < 70	14 (33%)	267 (49%)
• ≥ 70	28 (66%)	277 (51%)
BMI		
• < 18.5	4 (10%)	12 (2%)
• 18.5–24.9	15 (36%)	228 (42%)
• 25–29.9	13 (31%)	182 (33%)
• > 30	10 (24%)	122 (22%)
Weekly alcohol consumption		
• 0 units	11 (26%)	132 (24%)
• ≤ 14 units	24 (57%)	333 (61%)
• > 14 units	7 (17%)	79 (15%)
Smoking status		
• Non-smoker	10 (24%)	238 (44%)
• Ex-smoker	25 (60%)	220 (40%)
• Smoker	7 (17%)	86 (16%)
**Comorbidities**		
Hypertension	28 (67%)	253 (47%)
Myocardial infarction	7 (17%)	36 (7%)
Heart Failure	3 (7%)	16 (3%)
Atrial fibrillation	9 (21%)	53 (10%)
Peripheral artery disease	4 (10%)	23 (4%)
Cerebrovascular disease	6 (14%)	41 (8%)
Diabetes	13 (31%)	71 (13%)
**Preoperative state**		
ASA		
• 1–2	25 (61%)	436 (80%)
• 3–4	16 (39%)	107 (20%)
WHO Performance score		
• 0	22 (52%)	431 (79%)
• 1	15 (36%)	86 (16%)
• ≥ 2	5 (12%)	27 (5%)
UICC		
• 1–2	27 (64%)	290 (53%)
• 3–4	13 (31%)	228 (42%)
• missing	2 (5%)	26 (5%)
**Preoperative medication**		
Platelet inhibitors	16 (38%)	93 (17%)
Statins	18 (43%)	166 (31%)
Beta-Blockers	10 (24%)	78 (14%)
Diuretics	18 (43%)	108 (20%)
Calcium antagonists	11 (26%)	89 (16%)
ACE-Inhibitor/ARB	16 (38%)	165 (30%)
Aspirin	9 (21%)	84 (15%)
**Treatment**		
Neoadjuvant therapy	3 (7%)	32 (6%)
Laparoscopy	33 (79%)	420 (77%)
Robot-assisted laparoscopy	7 (17%)	116 (21%)
Laparotomy (from start or converted to)	6 (14%)	37 (7%)
Blood loss during surgery		
• < 100 ml	27 (64%)	416 (76%)
• 101–500 ml	12 (29%)	97 (18%)
• 501–999 ml	3 (7%)	17 (3%)
• > 1000 ml	0 (0%)	14 (3%)
**Postoperative course**		
Postoperative complications	18 (43%)	107 (20%)
Median LOS *(D)* [IQR]	7 (4–15)	4 (3–6)
Re-operation	8 (19%)	82 (15%)
Re-admission	4 (10%)	65 (12%)
ICU admission	8 (19%)	38 (7%)

*Baseline characteristics of the population undergoing surgery for colorectal cancer from June 2015 to July 2017 at Zealand University Hospital. Data are expressed as No. (%). Chi-square/Fishers Exact test or T-test/Wilcoxon Rank Sum test are performed*

*ACE Angiotensin converting enzyme, ARB Angiotensin receptor blocker, ASA American Society of Anaesthesiologist’s physical status classification system, BMI Body mass index, D days, ICU Intensive care unit, IQR Interquartile range, LOS Length of stay, MINS Myocardial injury after noncardiac surgery, UICC Union for International Cancer Control, WHO World Health Organization*

### All-cause mortality

**Fig. 1:**
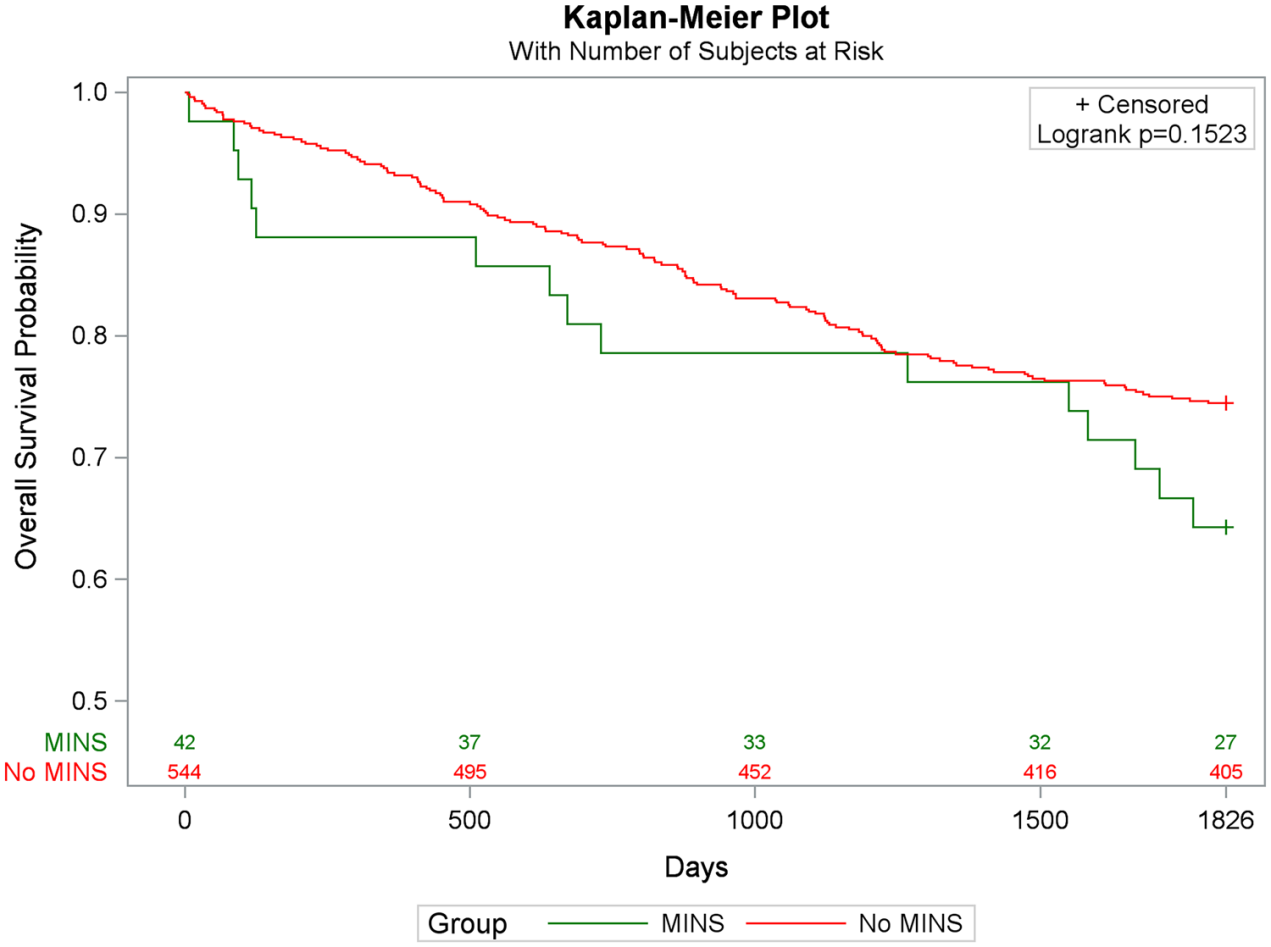
Kaplan–Meier Plot

During the first postoperative year, 12% of patients with MINS and 7% of patients without MINS died, p = 0.20. At postoperative year three, 21% with MINS and 18% without MINS had died, p = 0.58, and at postoperative year five 36% and 26% had died in the two groups, p = 0.15, respectively. The Kaplan–Meier plots showed a higher all-cause mortality in the patient group with MINS at all three time points, however, not statistically significant, see Fig. 1. When adjusted for age, sex, and UICC stage in the Cox regression model of one-year all-cause mortality, the adjusted HR for MINS was 2.40 (0.93 – 6.22). At year three and five, we additionally adjusted for ASA status, performance status, smoking and laparotomies, the adjusted HRs for MINS were 1.05 (0.51 – 2.15), and 1.11 (0.62 – 1.99), respectively. The unadjusted HRs for MINS are shown in Table 2.

**Table 2:** Hazard Ratios for all-cause mortality, recurrence, and disease-free survival in patients with myocardial injury

		**All-cause mortality**	**Recurrence**	**Disease-free survival**
		MINS	MINS	MINS
One year	HR (95% CI)	1.89 (0.74 – 4.82)	0.98 (0.13 – 7.61)	1.71 (0.73 – 4.01)
	aHR (95% CI)	2.40 (0.93 – 6.22)	1.47 (0.19 – 11.29)	2.25 (0.95 – 5.32)
Three years	HR (95% CI)	1.26 (0.64 – 2.50)	1.28 (0.45 – 3.64)	1.35 (0.74 – 2.46)
	aHR (95% CI)	1.05 (0.51 – 2.15)	1.38 (0.46 – 4.51)	1.19 (0.63 – 2.23)
Five years	HR (95% CI)	1.47 (0.86 – 2.50)	1.44 (0.57 – 3.66)	1.55 (0.95 – 2.53)
	aHR (95% CI)	1.11 (0.62 – 1.99)	1.49 (0.56 – 3.98)	1.19 (0.70 – 2.03)

*Unadjusted and adjusted hazard ratios for MINS. At year one the models are adjusted for sex, age groups and UICC stages and at year three and five the models are also adjusted for WHO performance status, ASA status, smoking status, and laparotomies.*

*aHR Adjusted Hazard ratio, HR hazard ratio, 95% CI 95% confidence interval*

### Recurrence

In the first postoperative year, the recurrence rate was the same for patients with MINS and those without MINS (2% vs. 3%, p = 1.00). Similarly, no difference at recurrence rate in patients with MINS and in those without were found at postoperative year three (10% and 8%, p = 0.77, respectively) nor at postoperative year five (12% and 9%, p = 0.58, respectively). The adjusted HR for MINS at one year was 1.47 (0.19 – 11.29), at three years 1.38 (0.46 – 4.51), and five years 1.49 (0.56 – 3.98). The unadjusted HRs for MINS were all statically non-significant, see Table 2. Furthermore, the competing risk model proposed by Fine and Gray showed similar associations – data not presented.

### Disease-free survival

When adjusting for age, sex, and UICC stage in a Cox regression model of either recurrence or mortality, the adjusted HR for the MINS group at one year was 2.25 (0.95 – 5.32), at three years 1.19 (0.63 – 2.23), and five years 1.19 (0.70 – 2.03). The unadjusted HRs for MINS are presented in Table 2 and shows a non-significant decreased risk of disease-free survival.

## Discussion

This retrospective cohort study included patients with troponin measurements after surgery for colorectal cancer in an ERAS setting and patients were followed for five years. Patients with MINS had an increased all-cause mortality and decreased disease-free survival at one, three, and five years after surgery; however, the absolute differences in all-cause mortality and disease-free survival between groups did not reach statistical difference. Recurrence rates were similar for patients with and without MINS.

Throughout the adjusted analyses for all-cause mortality, recurrence, and disease-free survival, patients with MINS had higher HRs compared to those without MINS, but none were statistically significant. Patients with UICC stage 3 and 4 had significantly higher HRs for all-cause mortality, recurrence, and disease-free survival in all adjusted analyses. In the same cohort, we have previously shown no significant difference in 90-day mortality between the MINS group and non-MINS group [12]. In the previous paper, we found that patients with MINS had a higher incidence of postoperative complications. Additionally, we observed that a higher POSPOM score was associated with an increased risk of MINS, and MINS was associated with a higher likelihood of postoperative complications [12].

Bleeding, hypoxemia, and hypotension are all associated with surgery [29–31], and in combination with the surgical stress response, it may lead to an oxygen supply–demand mismatch in the myocardium resulting in ischemic myocardial injury [11, 30, 31]. Furthermore, surgery induces acute endothelial dysfunction in the early postoperative period, which could add to the risk of myocardial injury [32–34]. Studies have shown that having a cancer is independently associated with a significantly increased risk for cardiovascular morbidity [15]. The other way around, it has also been shown that patients with cardiovascular disease have a higher risk of cancer than the general population [16]. One study has shown that low-grade inflammation is a risk factor for the development of cancer in patients with cardiovascular disease [35]. This implies that inflammation may be a shared underlying factor between cancer and cardiovascular disease.

In a Korean study, elevated high-sensitivity C-reactive protein concentrations at discharge appeared to be associated with increased long-term mortality in patients with MINS [36]. Other studies have shown that in patients with MINS, nonelective surgery is associated with long-term mortality [8]. Interestingly, in patients undergoing orthopedic surgery, trauma surgery was a predictor for long-term mortality after MINS [37]. In patients undergoing open radical cystectomy, there was a significantly lower one-year survival in patients with MINS compared to those without MINS (29% vs 12%) [38]. Elevated C-reactive protein concentrations itself indicate systemic inflammation [39], whereas non-elective/trauma surgery and open surgery all induce a larger surgical stress response causing more inflammation [40, 41]. The use of ERAS reduces postoperative inflammation following colorectal cancer surgery [42] and in our cohort the majority of the patients underwent minimally invasive surgery in an ERAS setting. Reduced postoperative inflammation might explain why we do not find any significant difference in short- or long-term mortality between patients with and without MINS compared to the findings of other studies with more prominent results [13, 43, 44]. However most importantly, our cohort has a low number of patients and limited statistical power to detect meaningful differences between the exposed and unexposed groups, even if a true association between MINS and long-term oncological outcomes exists. Our findings suggest a trend towards an increased risk of mortality and recurrence, which is consistent with the hypothesis of MINS negatively affecting the trajectory of a patient with cancer undergoing surgery. To our knowledge, no other studies have investigated associations between MINS and long-term oncological outcomes in patients undergoing colorectal cancer surgery, but in a small study of patients with head and neck cancer, no difference in three-year overall survival was found in patients with and without postoperative troponin elevations [45].

Our study is a retrospective study with both strengths and limitations. Firstly, unmeasured confounding is inevitable. Secondly, the cohort itself is relatively small and only 42 out of 586 patients developed MINS. During the study period, patients that had less than two troponin measurements were excluded, affecting the cohort size and the power of the study. The 2022 guidelines from European Society of Cardiology recommend to measure troponin concentration before intermediate- and high-risk noncardiac surgery and again at 24 h and 48 h after surgery in patients with cardiovascular disease or symptoms suggestive of it or with cardiovascular risk factors including age 65 or above [46]. Due to the nature of the retrospective study and the limited time-period in which troponin screening was performed at the department, we did not perform a sample size calculation prior to the study, and the study might not be adequately powered to determine significance in terms of long-term outcomes. Even though we did not have any missing data on outcomes of interest, we did have missing data on variables that could have been of interest such as details of the anesthetic techniques, drugs and peroperative monitoring. Furthermore, the limited sample size also restricts the number of covariates that can be adjusted for in a statistical analysis. On the other hand, the study also has several strengths. It examined a homogenous population following the ERAS protocol, ensuring the treatment of all patients was equally good and beneficial. In addition, a complete follow-up was performed on all patients, reducing selection bias and risk of misclassification of the exposure.

In conclusion, the absolute difference in long-term all-cause mortality between patients with and without MINS did not reach statistical significance. In both univariate and adjusted analyses, hazards ratios for all-cause mortality indicated an increased risk for patients with MINS but were not significant. Similarly, the hazards ratios for recurrence were not significant. The trends in colorectal cancer warrants greater focus on identifying patients that may be at higher risk of developing postoperative complications and/or early recurrence. Further studies exploring the association between MINS and long-term oncological outcomes in patients undergoing surgery for colorectal cancer is thus greatly needed. The findings of this study need to be supported by larger cohort studies with sufficient statistical power.

## Data Availability

Due to Danish and European Data Protection Regulations and due to the fact, that the individual patients of this study have not give written consent for their data to be shared publicly, supporting data is not available.
